# Longitudinal immune profiling demonstrates persistent MAIT cell reduction and temporal cytokine alterations after aneurysmal subarachnoid hemorrhage

**DOI:** 10.3389/fneur.2026.1900084

**Published:** 2026-08-20

**Authors:** Akos Merei, Noemi Balazs, Brotzki Da Costa Chayeen, Peter Engelmann, Timea Berki, Szabina Erdő-Bonyár, Diana Simon, Tihamer Molnar, Csaba Olah, Peter Csecsei

**Affiliations:** 1 Department of Anaesthesiology and Intensive Care, University of Pécs, Medical School, Pécs, Hungary; 2 Department of Immunology and Biotechnology, University of Pécs, Medical School, Pécs, Hungary; 3 Department of Neurosurgery, Borsod-Abaúj-Zemplén County Center Hospital and University Teaching Hospital, Miskolc, Hungary; 4 Department of Neurosurgery, University of Pécs, Medical School, Pécs, Hungary

**Keywords:** aneurysmal subarachnoid hemorrhage, inflammation, interleukins, mucosal-associated invariant T cells, outcome

## Abstract

**Background:**

Neuroinflammation and systemic immune dysregulation are increasingly recognized as key contributors to secondary brain injury after aneurysmal subarachnoid hemorrhage (aSAH). However, the temporal relationship between circulating cytokine responses and innate-like lymphocyte populations, particularly mucosal-associated invariant T (MAIT) cells, remains poorly characterized.

**Methods:**

In this prospective observational study, peripheral blood cytokine profiles were longitudinally analyzed in 48 patients with aSAH on days 1, 5, and 9 after ictus and compared with healthy controls. Serum concentrations of IL-6, IL-7, IL-12p40, IL-12p70, IL-15, IL-17A, IL-18, IL-22, IP-10, and CXCL-9 were measured using multiplex immunoassay. Flow cytometric immunophenotyping was performed in a subgroup of 16 patients to evaluate MAIT cells, NK cells, γδ T cells, iNKT cells, and NKT-like cells. Associations between immune parameters and 3-month functional outcome were also assessed.

**Results:**

Patients with aSAH demonstrated persistently elevated IL-6, IL-18, and IL-15 levels across all examined time points, while IL-7, IL-12p40, IP-10, and CXCL-9 showed delayed increases during the subacute phase. IL-6 and IL-15 concentrations were significantly higher in patients with unfavorable outcome, whereas IL-22 levels were increased in patients with favorable recovery. However, these associations were not independent of disease severity after adjustment for WFNS score and age. Flow cytometry revealed a persistent reduction in circulating MAIT-cell frequencies following aSAH, while other innate-like lymphocyte populations remained largely unchanged. DN MAIT-cell frequencies demonstrated significant negative correlations with circulating IL-18 levels across all examined time points.

**Conclusion:**

aSAH is associated with sustained systemic inflammatory activation and selective alterations of MAIT-cell populations. Although several cytokines were associated with functional outcome in univariate analyses, these associations were attenuated after adjustment for WFNS score and age, suggesting that they primarily reflect disease severity rather than independent prognostic effects. The observed relationship between MAIT-cell dysregulation and IL-18–associated inflammation provides further evidence of altered innate-like immune responses following aSAH.

## 1 Introduction

Aneurysmal subarachnoid hemorrhage (aSAH) is a devastating neurological emergency associated with high mortality and long-term disability despite advances in neurocritical care and aneurysm treatment (1). Subarachnoid hemorrhage (SAH) accounts for approximately 5–10% of all stroke cases, and nearly 85% of non-traumatic SAH events are caused by rupture of an intracranial aneurysm (2). According to recent Global Burden of Disease estimates, SAH was responsible for approximately 700,000 incident cases, 350,000 deaths, and more than 10 million disability-adjusted life years (DALYs) worldwide in 2021, highlighting its substantial global health burden (2). Current clinical guidelines emphasize the importance of rapid diagnosis, early aneurysm securing, and comprehensive neurocritical care management in order to reduce secondary complications and improve outcome (1). Nevertheless, clinical outcome after aSAH is not determined solely by the initial hemorrhagic insult, but also by complex secondary injury mechanisms including neuroinflammation, cerebral vasospasm, delayed cerebral ischemia, and systemic immune dysregulation (3).

Accumulating evidence suggests that systemic and central nervous system inflammation play a pivotal role in the pathophysiology of aSAH, contributing not only to early brain injury but also to delayed secondary complications and unfavorable neurological outcome (4, 5). Following aneurysm rupture, blood products and damage-associated molecular patterns (DAMPs) trigger a profound sterile inflammatory response. This response is characterized by activation of resident microglia, endothelial dysfunction, leukocyte recruitment, and disruption of the blood–brain barrier (5). Both experimental and clinical studies have demonstrated marked alterations in circulating inflammatory mediators after aSAH, including elevated levels of interleukins and chemokines associated with innate and adaptive immune activation (4, 6). Among these mediators, IL-6 has consistently emerged as one of the most prominent cytokines associated with disease severity, delayed cerebral ischemia, vasospasm, and poor clinical outcome after aSAH (4, 6). Increased IL-18 signaling has also been implicated in post-hemorrhagic neuroinflammation through inflammasome activation and amplification of innate immune responses (5). In parallel, chemokines involved in leukocyte trafficking, including interferon-inducible protein-10 (IP-10/CXCL-10) and CXCL-9, have been linked to persistent systemic inflammatory activation and recruitment of immune effector cells following hemorrhagic brain injury (4, 6). Increasing data support that cytokines involved in lymphocyte homeostasis and T-cell activation, such as IL-7, IL-12, IL-15, IL-17A, and IL-22, may also contribute to post-aSAH immune dysregulation, although their temporal dynamics and clinical relevance remain insufficiently characterized (4, 7). In addition to excessive inflammatory activation, aSAH is frequently accompanied by profound systemic immune alterations characterized by peripheral immunodepression, lymphocyte dysfunction, and increased susceptibility to infectious complications (7). Previous studies have demonstrated early reductions in circulating lymphocyte populations and impaired immune cell function after aSAH, suggesting a complex biphasic immune response involving simultaneous hyperinflammation and immunosuppression (7). Despite growing recognition of neuroimmune interactions after aSAH, the relationship between circulating inflammatory mediators and adaptive immune-cell alterations remains incompletely understood. Recent studies demonstrated dynamic changes in circulating T-cell populations and immune-cell activation following hemorrhage, suggesting that adaptive immunity may contribute to delayed cerebral ischemia, vascular dysfunction, and secondary brain injury after aSAH (8–11). Histopathological studies have demonstrated lymphocyte infiltration within aneurysm walls, while flow cytometric analyses identified dynamic alterations of circulating and cerebrospinal fluid T-cell populations after aSAH (8). Recent evidence suggests that adaptive immune responses contribute substantially to the pathophysiology of aSAH. Beyond innate immune activation, T lymphocytes have been implicated in post-hemorrhagic neuroinflammation, delayed cerebral ischemia, and vascular dysfunction following aneurysm rupture (8, 9). Previous studies demonstrated dynamic alterations in circulating T-cell populations and T-cell receptor repertoire diversity after aSAH, supporting the concept of complex adaptive immune activation during disease progression (8–11). Mucosal-associated invariant T (MAIT) cells are an innate-like T-cell subset characterized by semi-invariant T-cell receptors recognizing microbial metabolite antigens presented by MR1 (12). Beyond their role in antimicrobial immunity, MAIT cells have increasingly been implicated in sterile inflammatory and neuroimmune disorders (13). Recent studies suggest that MAIT cells may participate in central nervous system inflammatory responses through production of pro-inflammatory cytokines such as IL-17, IFN-*γ*, and TNF-*α*, as well as through interactions with monocytes, macrophages, and endothelial cells (13, 14). In ischemic stroke, circulating MAIT cell frequencies were shown to decrease rapidly after symptom onset, while activated MAIT cells accumulated within ischemic brain tissue, suggesting peripheral recruitment to sites of neuroinflammation (15). Experimental studies further demonstrated that MAIT-cell–derived IL-17 contributes to post-ischemic neuroinflammatory injury and neurological deterioration (14, 15). Given the substantial overlap between inflammatory mechanisms involved in ischemic and hemorrhagic brain injury, these findings raise the possibility that MAIT cells may also contribute to immune responses following aSAH. Despite growing recognition of the immunological importance of MAIT cells in neurological disorders, their role in aSAH remains largely unexplored.

Given the emerging role of neuroimmune interactions in aSAH, a more comprehensive characterization of systemic inflammatory mediators and innate-like lymphocyte populations may improve understanding of post-hemorrhagic immune dysregulation. In the present study, we performed longitudinal analysis of circulating cytokine profiles in patients with aSAH and combined these measurements with flow cytometric characterization of innate-like lymphocyte populations, including MAIT cells. We further investigated potential associations between inflammatory cytokines, immune-cell alterations, and clinical outcome in order to better characterize systemic immune dysregulation following aneurysmal subarachnoid hemorrhage. By integrating longitudinal cytokine measurements with cellular immune profiling, this study sought to provide further insight into the immunological mechanisms underlying secondary brain injury and systemic immune dysregulation after aneurysmal subarachnoid hemorrhage.

## 2 Methods

### 2.1 Study design and participants

Institutional ethical approval (IV/8468–1/2021/EKU and BM/4629–1/2024) for this prospective observational study was obtained prior to patient enrollment, and written informed consent was secured from all participants or their legal representatives before inclusion. Consecutive adult patients with aSAH admitted to our tertiary neurocritical care center were enrolled during the predefined study period. The diagnosis of aSAH was established by non-contrast cranial computed tomography (CT), with aneurysm confirmation by computed tomography angiography (CTA) or digital subtraction angiography (DSA). Only patients admitted within 24 h after ictus were included. For cytokine analyses, a total of 48 patients with aSAH were enrolled. Peripheral blood samples were collected longitudinally at three predefined time points: Day 1 (D1; 24 h after ictus), Day 5 (D5), and Day 9 (D9). Age- and sex-matched healthy individuals served as controls for cytokine measurements (*n* = 20). Healthy controls had no known acute or chronic inflammatory, autoimmune, infectious, or malignant disease at the time of sampling. Flow cytometric analyses were performed in a subgroup of 16 patients with aSAH and 12 healthy controls. Clinical, demographic, laboratory, and radiological data were prospectively collected for all enrolled patients. Recorded variables included age, sex, vascular risk factors (hypertension, diabetes mellitus, smoking history), aneurysm location, admission laboratory parameters, neurocritical care interventions, DCI, and 3-month functional outcome. Given the exploratory and hypothesis-generating nature of the study, no formal *a priori* sample size calculation was performed. The study was designed as an exploratory longitudinal investigation focusing on temporal alterations of systemic inflammatory mediators and innate-like lymphocyte populations following aSAH. Patients with active systemic infection, known autoimmune or renal disease, chronic immunosuppressive treatment, hematological malignancy, or recent major surgery or trauma were excluded whenever applicable. The patient screening and enrollment process is summarized in the STROBE flow diagram (Supplementary Figure S1).

### 2.2 Clinical definitions and outcome assessment

Neurological severity at admission was assessed using the World Federation of Neurosurgical Societies (WFNS) scale. Admission laboratory parameters included serum creatinine, C-reactive protein (CRP), white blood cell (WBC) count, and neutrophil-to-lymphocyte ratio (NLR). Neurocritical care interventions, including mechanical ventilation and extraventricular drain (EVD) placement, were recorded throughout hospitalization. DCI was diagnosed based on established consensus criteria as the occurrence of new neurological deterioration and/or new cerebral infarction not attributable to alternative causes such as rebleeding, hydrocephalus, metabolic disturbances, seizures, or procedural complications (16). Functional outcome was assessed at 3 months using the modified Rankin Scale (mRS). Favorable outcome was defined as mRS 0–2, while unfavorable outcome was defined as mRS 3–6. These outcome categories were used for all comparative analyses of cytokine concentrations and immune cell populations.

### 2.3 PBMC isolation

Peripheral blood mononuclear cells (PBMCs) were isolated from whole blood using Ficoll density-gradient centrifugation. Briefly, blood samples collected in EDTA tubes (Greiner Bio-One, Austria) were diluted 1:1 with phosphate-buffered saline (PBS) and carefully layered over Ficoll-Paque™ (Cytiva, USA). Samples were centrifuged at 733 × g for 20 min at room temperature without brake. The PBMC layer at the plasma–Ficoll interface was collected and transferred to a new tube. Cells were washed twice with PBS by centrifugation at 183 × g for 10 and 5 min to remove residual Ficoll and platelets. The final cell pellet was resuspended in PBS containing 0.1% (w/v) bovine serum albumin and sodium azide. Cell count and viability were determined using trypan blue exclusion with a LUNA-II™ Automated Cell Counter (Logos Biosystems, South Korea). PBMC isolation and flow cytometric staining were performed within 2 h after blood collection.

### 2.4 Flow cytometry analysis

Flow cytometric analyses were performed in a subset of 16 patients with aSAH and 12 healthy controls. The healthy flow cytometry controls represented a subset of the 20 healthy individuals included in the cytokine cohort and were selected based on the availability of fresh PBMC samples suitable for cellular analyses. The flow cytometry control subgroup was independently compared with the corresponding patient subgroup with respect to age and sex. Flow cytometry data analysis was performed by investigators blinded to the patients’ clinical characteristics and outcome. Freshly isolated PBMCs were used for flow cytometric analysis. Briefly, 1 × 10^6^ cells per tube were stained with fluorochrome-conjugated anti-human monoclonal antibodies in round-bottom polystyrene tubes for 30 min at room temperature in the dark. Following staining, cells were washed with staining buffer by centrifugation at 183 × g for 10 min and resuspended in PBS containing 1% paraformaldehyde. Data acquisition was performed immediately using a DxFLEX™ flow cytometer (Beckman Coulter Inc., USA). Compensation was calculated using CytExpert™ software (Xitogen Technologies, Suzhou, China) based on single-stained PBMC samples. Because flow cytometric analysis required immediate processing of freshly isolated peripheral blood mononuclear cells (PBMCs), inclusion in the flow cytometry subgroup depended on the availability of trained laboratory personnel and the required laboratory infrastructure at the time of the first blood sampling. Consequently, patients whose initial blood sample was obtained during routine laboratory operating hours on weekdays (approximately 06:00–18:00) were eligible for flow cytometric analysis. Once enrolled, all subsequent scheduled samples (Days 5 and 9) were prospectively processed irrespective of the day of collection. Selection was therefore based on logistical feasibility rather than clinical characteristics or sample quality.

### 2.5 Antibodies

The following anti-human monoclonal antibodies were used for surface staining: anti-CD45 Alexa Fluor 700 (AF700; clone HI30), anti-CD3 allophycocyanin (APC; clone UCHT1), anti-CD4 APC-Cy7 (clone A161A1), anti-CD8 Brilliant Violet 605 (BV605; clone SK1), anti-CD56 PE-Cy7 (clone MEM-188), anti-CD161 PerCP-Cy5.5 (clone HP-3G10), anti-TCRγδ BV421 (clone B1), anti-TCRVα7.2 FITC (clone 3C10), and anti-TCRVα24-Jα18 PE (clone 6B11). All antibodies were purchased from BioLegend (USA).

### 2.6 Flow cytometry gating strategy

Flow cytometry data were analyzed using a sequential gating strategy. Lymphocytes were first identified based on forward scatter (FSC) and side scatter (SSC) properties, and debris was excluded. Doublets were removed by gating on FSC-A versus FSC-H parameters to ensure analysis of single cells. Subsequent gating was performed using fluorescence-conjugated antibodies specific to surface markers. T cells were defined as CD45^+^/CD3^+^ cells and further subdivided into CD4^+^ and CD8^+^ subsets. Natural killer (NK) cells were identified as CD3^−^/CD56^+^, and NKT-like cells as CD3^+^/CD56^+^. γδ T cells were defined as CD3^+^/TCRγδ^+^ cells. Mucosal-associated invariant T (MAIT) cells were identified within the CD3^+^ T-cell population as CD161^+^/TCRVα7.2^+^ cells, and invariant natural killer T (iNKT) cells as invariant natural killer T (iNKT) cells were identified within CD3^+^ T cells as CD161^+^/TCRVα24-Jα18^+^ cells.

### 2.7 Serum cytokine measurement

Serum samples were centrifuged immediately after collection, aliquoted, and stored at −80 °C until cytokine analysis. The serum concentration of IL-6, IL-7, IL-12p40, IL-12p70, IL-15, IL-17A, IL-18, IL-22, IP-10 and CXCL-9 were determined using MILLIPLEX Human Cytokine/Chemokine/Growth Factor Panel A Magnetic Bead Panel (HCYTA-60 K, Merck KGaA, Darmstadt, Germany) according to the manufacturer’s instructions. The assay was run with Luminex MAGPIX instrument (Luminex Corporation, Austin, TX, USA). Data were analyzed with Belysa Immunoassay Curve Fitting Software (Merck KGaA, Darmstadt, Germany). The selected sampling schedule (Days 1, 5, and 9) represented a predefined compromise between capturing the major phases of the early inflammatory response after aSAH and maintaining the feasibility of repeated blood collection within a prospective longitudinal biobanking protocol that supported multiple parallel translational studies.

### 2.8 Statistical analysis

Statistical analyses were performed using appropriate parametric and non-parametric methods depending on data distribution. Data distribution was assessed using the Shapiro–Wilk test and visual inspection of histograms. Continuous variables are presented as mean ± standard deviation (SD) or median with interquartile range (IQR), as appropriate, while categorical variables are expressed as counts and percentages. Comparisons between two independent groups (SAH vs. controls or favorable vs. unfavorable outcome) were performed using the Student’s *t*-test for normally distributed variables or the Mann–Whitney *U* test for non-normally distributed variables. Categorical variables were compared using the χ^2^ test or Fisher’s exact test, as appropriate. For cytokine analyses, differences between SAH patients and controls at each time point (Day 1, Day 5, Day 9) were assessed using the Mann–Whitney *U* test. For patient-versus-control cytokine comparisons, Mann–Whitney U tests were performed separately at each time point. To control for multiple testing, the resulting *p*-values from all 30 comparisons (10 cytokines measured at three time points) were adjusted using the Benjamini–Hochberg false discovery rate (FDR) procedure. FDR-adjusted *q*-values below 0.05 were considered statistically significant. Longitudinal changes in cytokine levels within the SAH cohort were analyzed using linear mixed-effects models applied to log-transformed cytokine concentrations. Time was included as a fixed effect, and patient ID as a random intercept to account for repeated measurements. To determine whether longitudinal cytokine trajectories differed according to functional outcome, additional linear mixed-effects models were constructed separately for IL-6, IL-15, and IL-22. Log-transformed cytokine concentrations were used as dependent variables, while time, outcome group (favorable vs. unfavorable), and the time-by-outcome group interaction were included as fixed effects. Patient ID was included as a random intercept to account for repeated measurements. Time was treated as a categorical variable, with Day 1 as the reference category. Because zero cytokine concentrations were present, values were transformed using log(1 + x). Log-transformation was applied only for linear mixed-effects modeling to improve model assumptions, whereas non-parametric analyses were performed using the original cytokine concentrations. The overall significance of the time-by-outcome interaction was evaluated by comparing models with and without the interaction term using likelihood-ratio tests. The resulting interaction *p*-values for the three cytokines were adjusted using the Benjamini–Hochberg false discovery rate procedure.

Associations between cytokine levels and clinical outcome were initially evaluated using univariate comparisons (Mann–Whitney *U* test) at each time point. To assess the independent association between early cytokine levels and 3-month functional outcome, multivariable Firth penalized logistic regression analyses were performed using Day 1 cytokine concentrations as predictors. Firth’s penalized likelihood approach was chosen to reduce small-sample bias associated with conventional maximum likelihood estimation. Models were adjusted for age and WFNS score. Results are presented as odds ratios (ORs) with 95% confidence intervals (CIs). Correlation analyses between cytokine levels and immune cell subsets were performed using Spearman’s rank correlation coefficient (*ρ*). To account for multiple testing, *p*-values obtained from the six Spearman correlation analyses between DN MAIT-cell frequencies and IL-6/IL-18 across the three study time points were adjusted using the Benjamini–Hochberg false discovery rate (FDR) procedure. Pairwise correlations across all cytokines and time points were visualized using a correlation matrix and hierarchical clustering based on similarity of correlation patterns. Flow cytometry data involving more than two groups (control, D1, D5, D9) were analyzed using the Kruskal–Wallis test followed by Dunn’s multiple comparisons test. Effect sizes for cytokine differences between SAH patients and controls were expressed as fold changes calculated from median values. All statistical tests were two-tailed, and a *p*-value <0.05 was considered statistically significant unless otherwise specified. Analyses were performed using available data without imputation. Statistical analyses were conducted using IBM SPSS Statistics (version 25), and figures were generated using GraphPad Prism (version 10.0.2).

## 3 Results

### 3.1 Patients characteristics

A total of 48 patients with aSAH and 20 healthy controls were included for cytokine analyses. No patients were lost during the predefined longitudinal follow-up, and serum samples were successfully obtained from all 48 patients at all three planned sampling time points (Days 1, 5, and 9). Flow cytometric analyses were performed in a representative subgroup of 16 patients and 12 controls. Demographic and baseline clinical characteristics are summarized in Table 1. No significant differences in age, sex distribution, or vascular risk factors were observed between patient and control groups. Clinical characteristics of the flow cytometry subgroup were comparable to those of the overall aSAH cohort. DCI occurred in 25% of SAH patients and 31% of the subgroup. Extraventricular drain placement was required in 50 and 44% of patients, while mechanical ventilation was used in 48 and 38%, respectively. Favorable outcome was observed in 44% of the overall SAH cohort and 50% of the flow cytometry subgroup, Table 1. Key clinical variables, including WFNS score, DCI occurrence, and outcome distribution, were comparable between the full cohort and the flow cytometry subgroup.

**Table 1:** Baseline demographic and clinical characteristics of the overall aSAH cohort and the flow cytometry subgroup.

Variable	SAH cohort (*n* = 48)	Control cohort (*n* = 20)	*p*-value	SAH flow subgroup (*n* = 16)	Control flow subgroup (*n* = 12)	*p*-value
Age (mean ± SD)	54 ± 11	50 ± 7	0.159	54 ± 10	48 ± 6	0.074
Female, *N* (%)	35 (72)	12 (60)	0.389	13 (81)	9 (75)	0.69
Hypertension, *N* (%)	28 (58)	10 (50)	0.597	9 (56)	3 (25)	0.098
Diabetes, *N* (%)	5 (10)	2 (10)	0.665	1 (6)	0 (0)	0.378
Smoking, *N* (%)	11 (23)	4 (20)	0.533	4 (25)	3 (25)	1.000
Anterior circulation aneurysm, N (%)	36 (75)	N/A	N/A	12 (75)	N/A	N/A
WFNS, median [IQR]	2 [1–4]	N/A	N/A	2 [2–3]	N/A	N/A
Creatinine*, median [IQR]	65 [58–79]	N/A	N/A	63 [57–71]	N/A	N/A
CRP*, median [IQR]	3 [2–7]	N/A	N/A	3.8 [1–11]	N/A	N/A
WBC*, median [IQR]	12.4 [10–17]	N/A	N/A	14 [11–18]	N/A	N/A
Neutrophil-to-lymphocyte ratio*, median [IQR]	5 [3–8]	N/A	N/A	5.9 [3–8]	N/A	N/A
Delayed cerebral ischemia, *N* (%)	12 (25)	N/A	N/A	5 (31)	N/A	N/A
Extraventricular drain, *N* (%)	24 (50)	N/A	N/A	7 (44)	N/A	N/A
Mechanical ventilation, *N* (%)	23 (48)	N/A	N/A	6 (38)	N/A	N/A
Favorable outcome, *N* (%)	21 (44)	N/A	N/A	8 (50)	N/A	N/A

SD, standard deviation; IQR, interquartile range; WFNS, World Federation of Neurosurgical Societies scale; CRP, C-reactive protein; WBC, white blood cell count; NS, not significant; N, number. Values marked with * were obtained on admission. *p*-values represent comparisons between SAH and control cohorts, and between SAH flow subgroup and control flow subgroup. Continuous variables were compared using Student’s *t*-test or Mann–Whitney U test, categorical variables using χ^2^ or Fisher’s exact test. The healthy controls included in the flow cytometry analyses (*n* = 12) represent a subset of the cytokine control cohort (*n* = 20).

### 3.2 Comparison of cytokine levels between aSAH patients and controls

**Figure 1:**
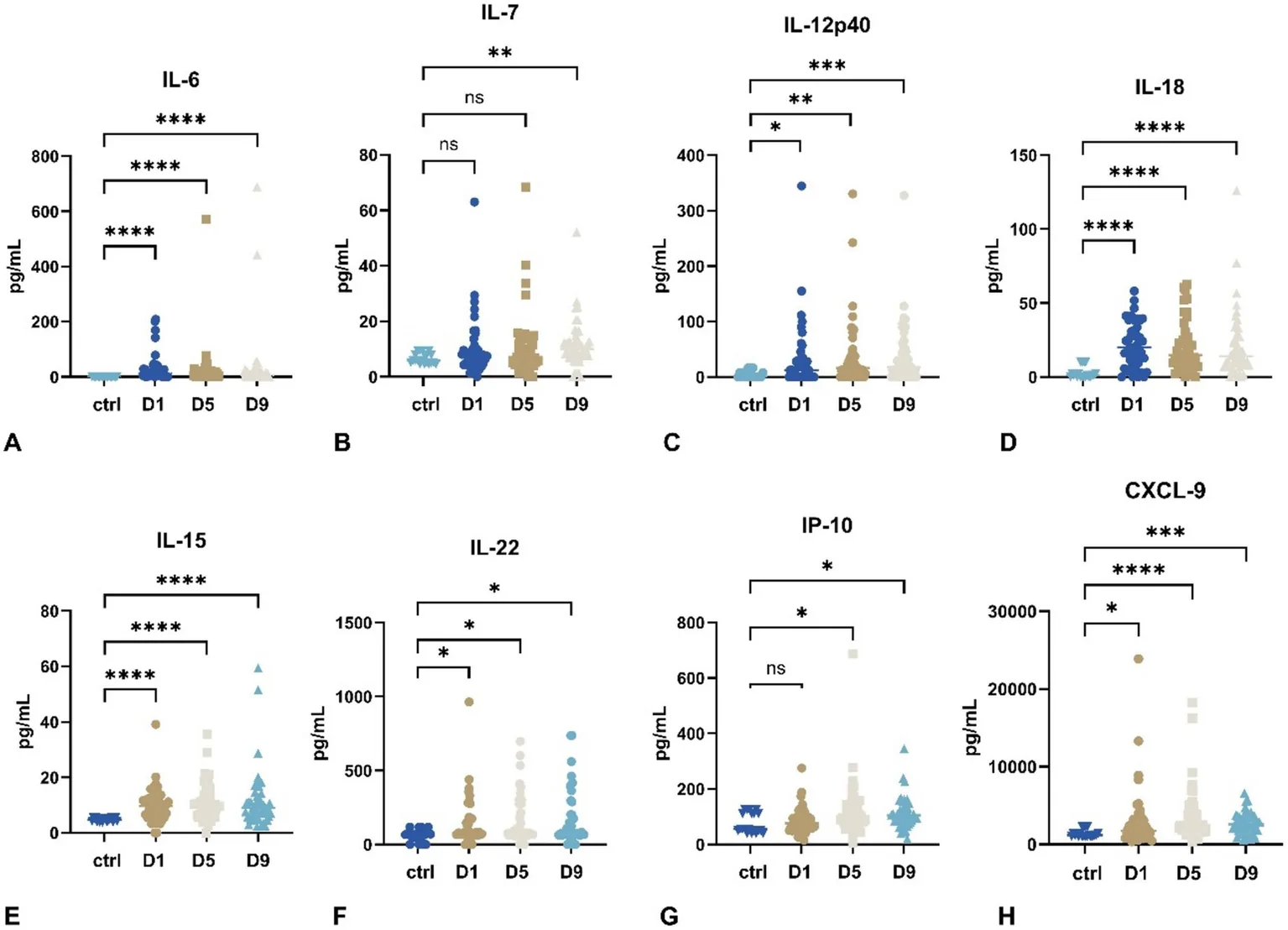
Comparison of temporal cytokine profiles in aneurysmal subarachnoid hemorrhage (aSAH) patients and healthy controls. Peripheral blood cytokine concentrations in patients with aneurysmal subarachnoid hemorrhage (aSAH, *n* = 48) measured on Day 1 (D1), Day 5 (D5), and Day 9 (D9) after ictus and compared with healthy controls (ctrl, *n* = 20). Cytokine concentrations are expressed in pg./mL, and each data point represents one individual participant. Statistical comparisons between each patient time point and controls were performed using two-sided Mann–Whitney U tests with Benjamini–Hochberg FDR correction for multiple testing (30 comparisons). Significance is reported as FDR-adjusted *q*-values. **(A)** IL-6, **(B)** IL-7, **(C)** IL-12p40, **(D)** IL-18, **(E)** IL-15, **(F)** IL-22, **(G)** IP-10, and **(H)** CXCL-9. Significance levels: NS, not significant; *q* < 0.05; *q* < 0.01; *q* < 0.001; *q* < 0.0001. Exact FDR-adjusted *q*-values are provided in Supplementary Table S1.

Longitudinal cytokine dynamics within the aSAH cohort were evaluated using linear mixed-effects modeling, while comparisons between aSAH patients and healthy controls at individual time points were assessed using Mann–Whitney U tests followed by a global Benjamini–Hochberg false discovery rate (FDR) correction across all 30 comparisons. Serum IL-6, IL-18, and IL-15 levels were markedly elevated in aSAH patients compared to controls at all examined time points (D1, D5, D9; all *q* < 0.001; Figures 1A,D,E). These cytokines exhibited large effect sizes, with median fold increases ranging from approximately 10- to 25-fold for IL-6 and IL-18, and around 2-fold for IL-15. Despite this pronounced elevation, linear mixed-effects modeling (log-transformed values, random intercept for patient ID) revealed no significant temporal changes between D1, D5, and D9, indicating persistently elevated but stable levels over time. In contrast, IL-22 demonstrated a modest but consistent increase compared with controls across all time points (all *q* < 0.05; Figure 1F), with small effect sizes (~1.1-fold), and no significant temporal variation in mixed-effects analysis. IL-7 and IL-12p40 displayed distinct temporal patterns. IL-7 did not differ from controls at D1 or D5 but was significantly elevated at D9 (*q* = 0.0013; Figure 1B), accompanied by a trend toward increasing levels over time (D9 vs D1: *p* = 0.062). In contrast, IL-12p40 levels were already significantly higher than controls at D1 and remained elevated at D5 and D9 (all *q* < 0.05; Figure 1C). Longitudinal analysis further demonstrated a significant increase at D9, while a non-significant increase was observed at D5, indicating persistent elevation with additional temporal augmentation. A comparable pattern was observed for chemokines. IP-10 levels did not differ from controls at D1 but were significantly elevated at both D5 (*q* = 0.0117) and D9 (*q* = 0.0302; Figure 1G), while linear mixed-effects modeling confirmed a significant increase over time. Similarly, CXCL-9 concentrations were already significantly higher than controls at D1 (*q* = 0.037), with more pronounced differences at D5 (*q* < 0.001) and D9 (*q* < 0.001; Figure 1H). Longitudinal analysis demonstrated a significant increase at D5, while concentrations continued to numerically increase toward D9. Exact FDR-adjusted *q*-values are provided in Supplementary Table S1.

**Figure 2:**
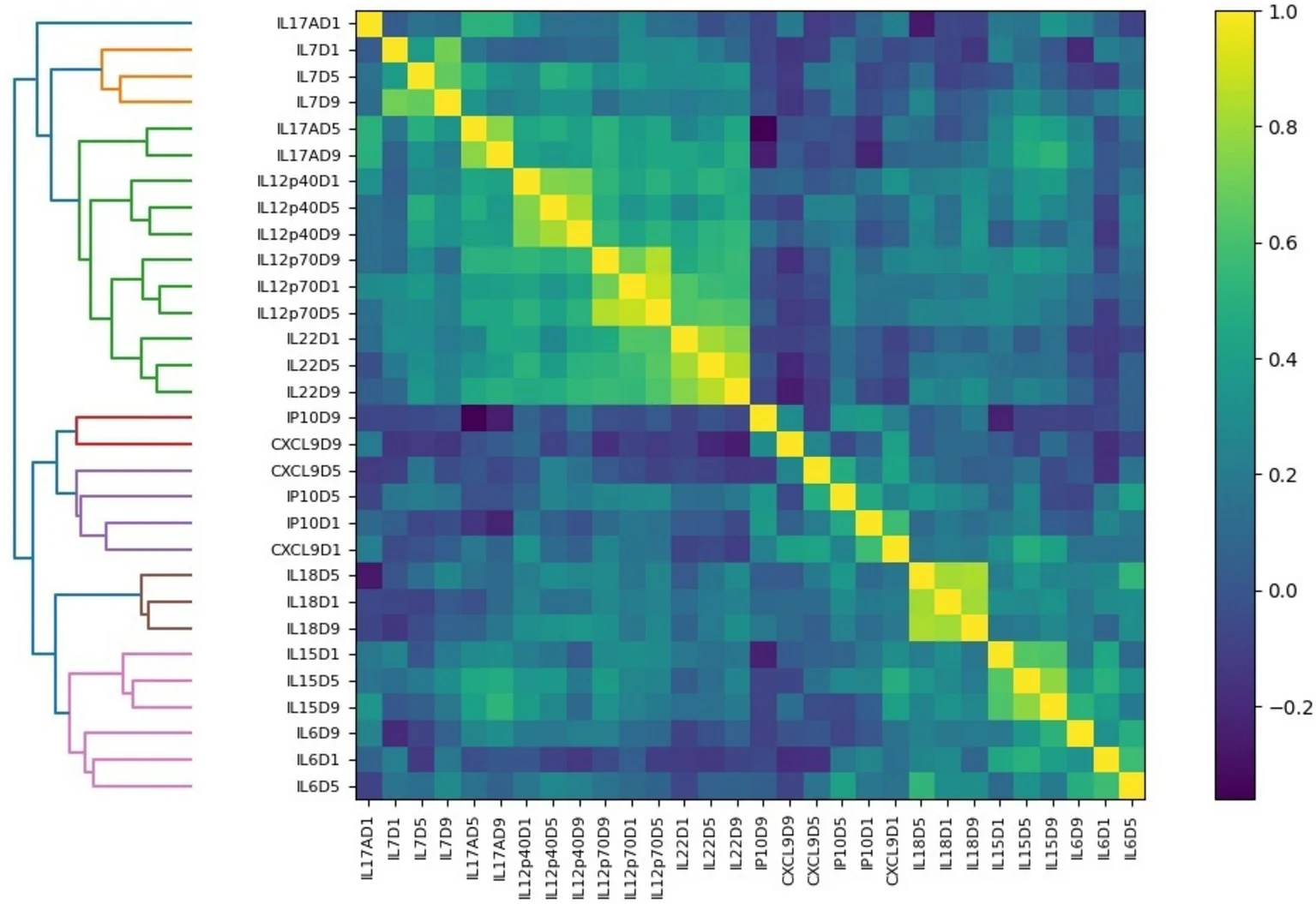
Correlation-based hierarchical clustering of circulating cytokines following aSAH. Spearman rank correlation coefficients were calculated pairwise between circulating cytokine levels measured in patients with aSAH (*n* = 48) across all available time points (Day 1 [D1], Day 5 [D5], and Day 9 [D9] after ictus). The resulting correlation matrix is visualized as a heatmap, with colors representing the strength and direction of correlations according to the color scale (from negative to positive values). Hierarchical clustering was applied to the correlation matrix to group cytokines based on shared correlation patterns, and is displayed as a dendrogram on the left side of the heatmap. Each variable represents a specific cytokine measured at a given time point (e.g., IL-6D1, IL-6D5, IL-6D9).

To explore relationships between inflammatory mediators and their temporal dynamics, pairwise Spearman correlation analysis of serum cytokine levels was performed across all measured time points and is presented as a heatmap in Figure 2. Hierarchical clustering revealed distinct groups of cytokines with similar correlation patterns. Measurements of the same cytokine across different time points showed strong positive correlations, particularly for IL-12p40, reflecting consistent temporal behavior. IL-12p40 measurements also demonstrated positive correlations with IL-22 and IL-7 across time points. A separate cluster was observed for IL-15 and IL-18, which were closely correlated with each other across time points, with IL-6 showing moderate associations within this group. In contrast, IP-10 and CXCL-9 formed a separate cluster characterized by strong within-cytokine correlations across time points and comparatively weaker correlations with interleukin measurements.

### 3.3 Association of serum cytokine levels with 3-month clinical outcome after aSAH

**Figure 3:**
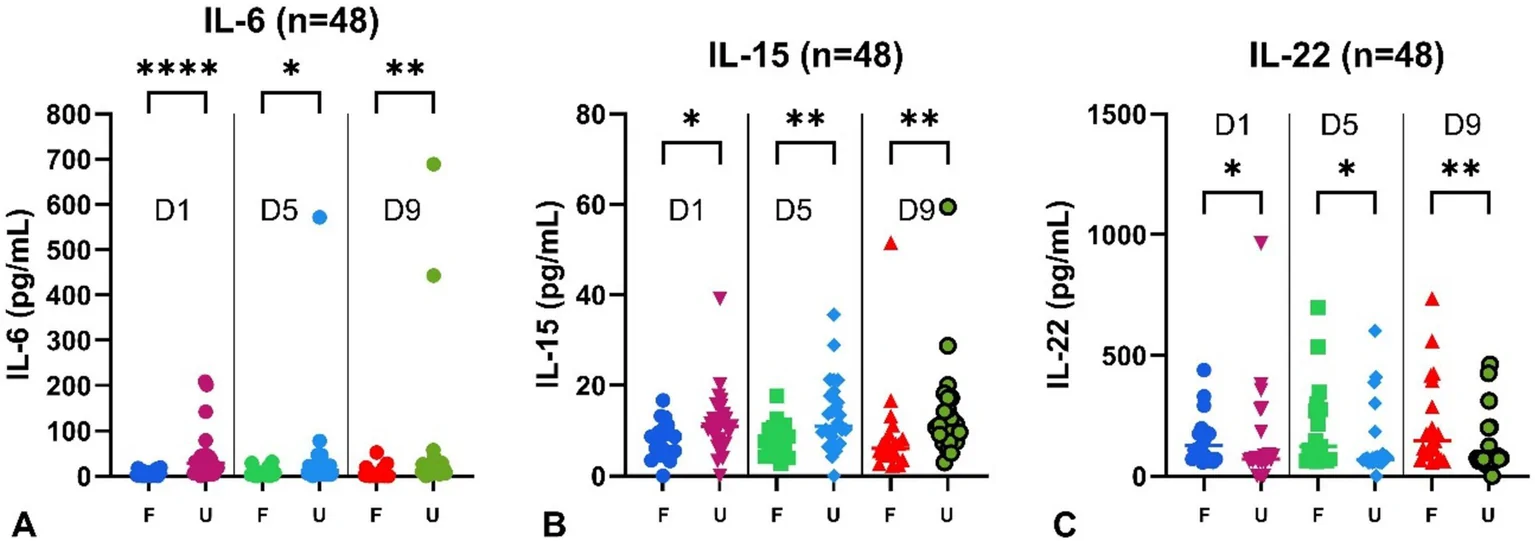
Association of cytokine levels with 3-month outcome in patients with aneurysmal subarachnoid hemorrhage (aSAH). Peripheral blood cytokine concentrations were measured in patients with aSAH (*n* = 48) at three time points: Day 1 (D1), Day 5 (D5), and Day 9 (D9) after ictus. Patients were stratified according to clinical outcome into favorable (F; mRS 0–2, *n* = 21) and unfavorable (U; mRS 3–6, *n* = 27) groups. Cytokine concentrations are expressed in pg./mL. Cytokine concentrations are expressed in pg./mL, and each symbol represents an individual participant. For each timepoint, bars correspond to favorable and unfavorable outcome groups. **(A)** IL-6, **(B)** IL-15, **(C)** IL-22. Statistical comparisons between groups at each timepoint were performed using the Mann–Whitney U test. Significance levels are indicated as follows: ns (not significant), *p < 0.05, **p < 0.01, ***p < 0.001, ****p < 0.0001. *n*, number; IL, interleukin; mRS, modified Rankin Scale.

Peripheral blood cytokine concentrations stratified by clinical outcome are shown in Figure 3. Patients were categorized into favorable (mRS 0–2, *n* = 21) and unfavorable (mRS 3–6, *n* = 27) outcome groups, with measurements obtained on days 1, 5, and 9 after ictus. IL-6 and IL-15 levels were consistently higher in patients with unfavorable outcome compared to those with favorable outcome across all examined time points, with statistically significant differences observed at D1, D5, and D9 (Figures 3A,B). In contrast, IL-22 levels showed an opposite pattern, with higher concentrations observed in the favorable outcome group at all time points, reaching statistical significance at D1, D5, and D9 (Figure 3C). Statistical comparisons between outcome groups at each time point were performed using the Mann–Whitney U test. To evaluate whether early cytokine concentrations were independently associated with 3-month functional outcome, multivariable Firth penalized logistic regression analyses were performed using Day 1 cytokine levels while adjusting for age and WFNS score. None of the investigated cytokines (IL-6, IL-15, or IL-22) reached statistical significance in these exploratory penalized regression models (Table 2), whereas WFNS score remained a significant predictor in all three models. Given the limited sample size and the restricted number of covariates that could be included without model overfitting, these findings should be interpreted cautiously and should not be considered definitive evidence against an independent association. To assess whether longitudinal cytokine trajectories differed according to functional outcome, additional linear mixed-effects models including time, outcome group, and their interaction were performed for IL-6, IL-15, and IL-22. No significant overall time-by-outcome interaction was identified for IL-6 (*p* = 0.415, FDR-adjusted *q* = 0.552), IL-15 (*p* = 0.552, *q* = 0.552), or IL-22 (*p* = 0.529, *q* = 0.552). Similarly, none of the individual Day 5 or Day 9 interaction coefficients reached statistical significance. Thus, although cytokine concentrations differed between favorable and unfavorable outcome groups in the univariate comparisons at individual time points, their longitudinal trajectories between Days 1 and 9 were broadly parallel.

**Table 2:** Firth penalized logistic regression analyses of Day 1 cytokine levels and 3-month functional outcome after aneurysmal subarachnoid hemorrhage.

Model	Predictor	OR	95% CI	*p*-value
IL-6 model	IL-6	1.011	0.986–1.035	0.392
	WFNS	3.16	1.54–6.47	0.0017
	Age	1.034	0.965–1.107	0.342
IL-15 model	IL-15	1.098	0.942–1.280	0.232
	WFNS	3.45	1.71–6.97	0.00054
	Age	1.028	0.958–1.102	0.444
IL-22 model	IL-22	1.002	0.998–1.006	0.375
	WFNS	3.86	1.88–7.90	0.00022
	Age	1.039	0.971–1.112	0.264

Multivariable Firth penalized logistic regression analyses evaluating the association between Day 1 serum cytokine concentrations (IL-6, IL-15, and IL-22) and 3-month functional outcome following aneurysmal subarachnoid hemorrhage (aSAH). Separate models were constructed for each cytokine and adjusted for age and admission World Federation of Neurosurgical Societies (WFNS) score. Results are presented as odds ratios (ORs), 95% confidence intervals (CIs), and corresponding *p*-values. Firth’s penalized likelihood approach was used to reduce small-sample bias associated with conventional maximum likelihood estimation.

### 3.4 Temporal changes in peripheral lymphocyte subsets following aSAH

**Figure 4:**
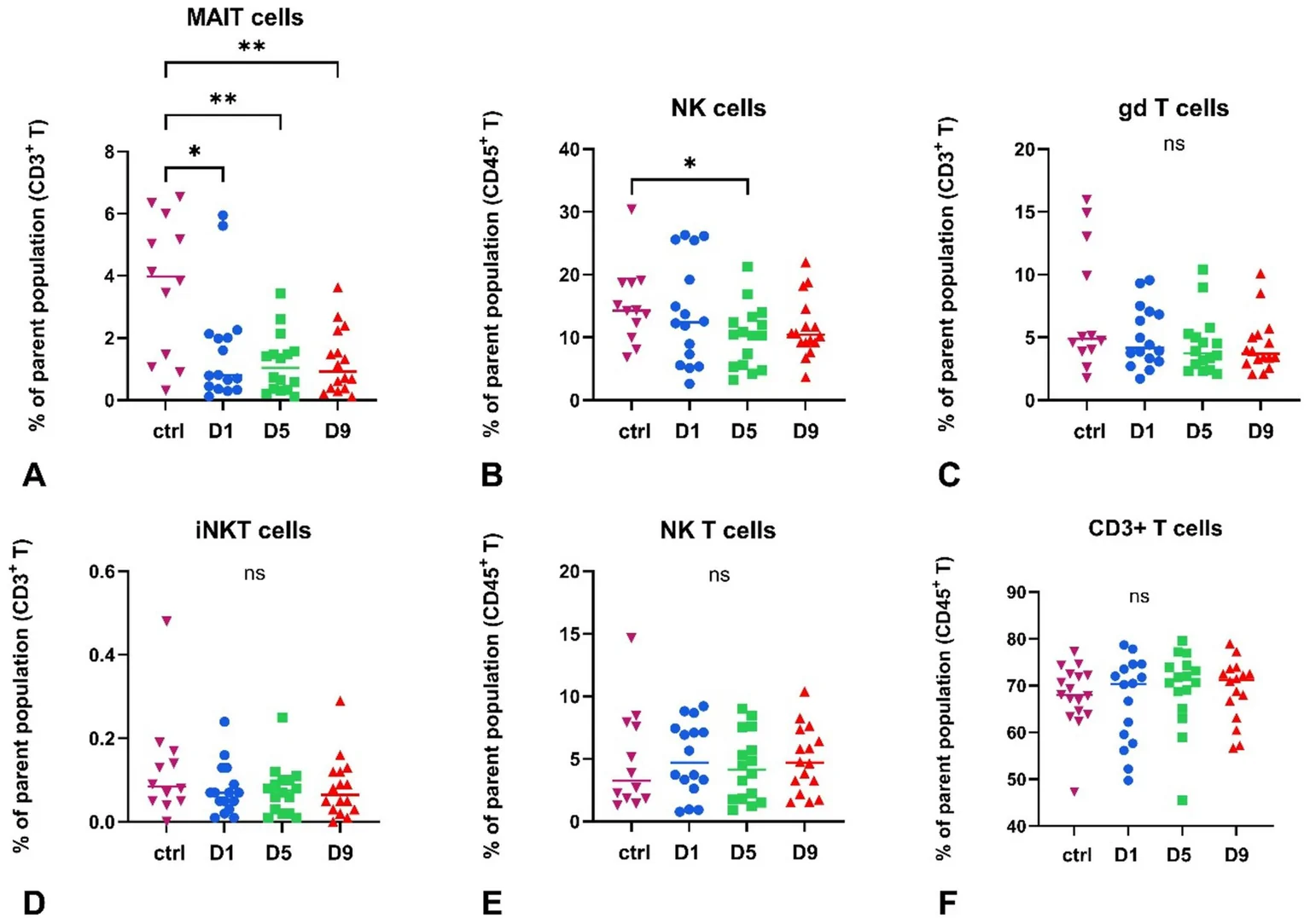
Longitudinal analysis of circulating lymphocyte subsets following aSAH. Flow cytometry was performed in a subset of patients with aSAH (*n* = 16) and compared to healthy controls (ctrl, *n* = 12). Peripheral blood samples were collected at day 1 (D1), day 5 (D5), and day 9 (D9) after ictus. Cell populations are expressed as percentages of their respective parent populations. Data are presented as individual value plots, with each symbol representing one participant. **(A)** MAIT cells (mucosal-associated invariant T cells; % of CD3^+^ T cells). **(B)** NK cells (natural killer cells; % of CD45^+^ lymphocytes). **(C)** γδ T cells (gamma-delta T cells; % of CD3^+^ T cells). **(D)** iNKT cells (invariant natural killer T cells; % of CD3^+^ T cells). **(E)** NKT-like cells (natural killer T-like cells; % of CD45^+^ lymphocytes). **(F)** CD3^+^ T cells (total T lymphocytes; % of CD45^+^ lymphocytes). Statistical comparisons between groups were performed using the Kruskal–Wallis test followed by Dunn’s multiple comparisons test. Significance levels are indicated as follows: s (not significant), *p < 0.05, **p < 0.01.

Flow cytometric analysis of peripheral blood lymphocyte subsets in the aSAH flow cytometry subgroup is shown in Figure 4. MAIT cell frequencies were significantly reduced compared with controls at all examined time points, with differences observed at D1 (*p* < 0.05), D5 (*p* < 0.01), and D9 (*p* < 0.01; Figure 4A). NK cell proportions differed between groups, with a significant reduction observed at D5 compared to controls (*p* < 0.05; Figure 4B), while no differences were detected at D1 or D9. In contrast, no significant differences were observed for γδ T cells, iNKT cells, or NKT like cells between controls and aSAH patients at any time point (Figures 4C–E). Similarly, the proportion of total CD3^+^ T cells within the CD45^+^ lymphocyte population remained unchanged across all examined groups (Figure 4F).

### 3.5 Alterations in MAIT cell subsets and their association with circulating cytokines

**Figure 5:**
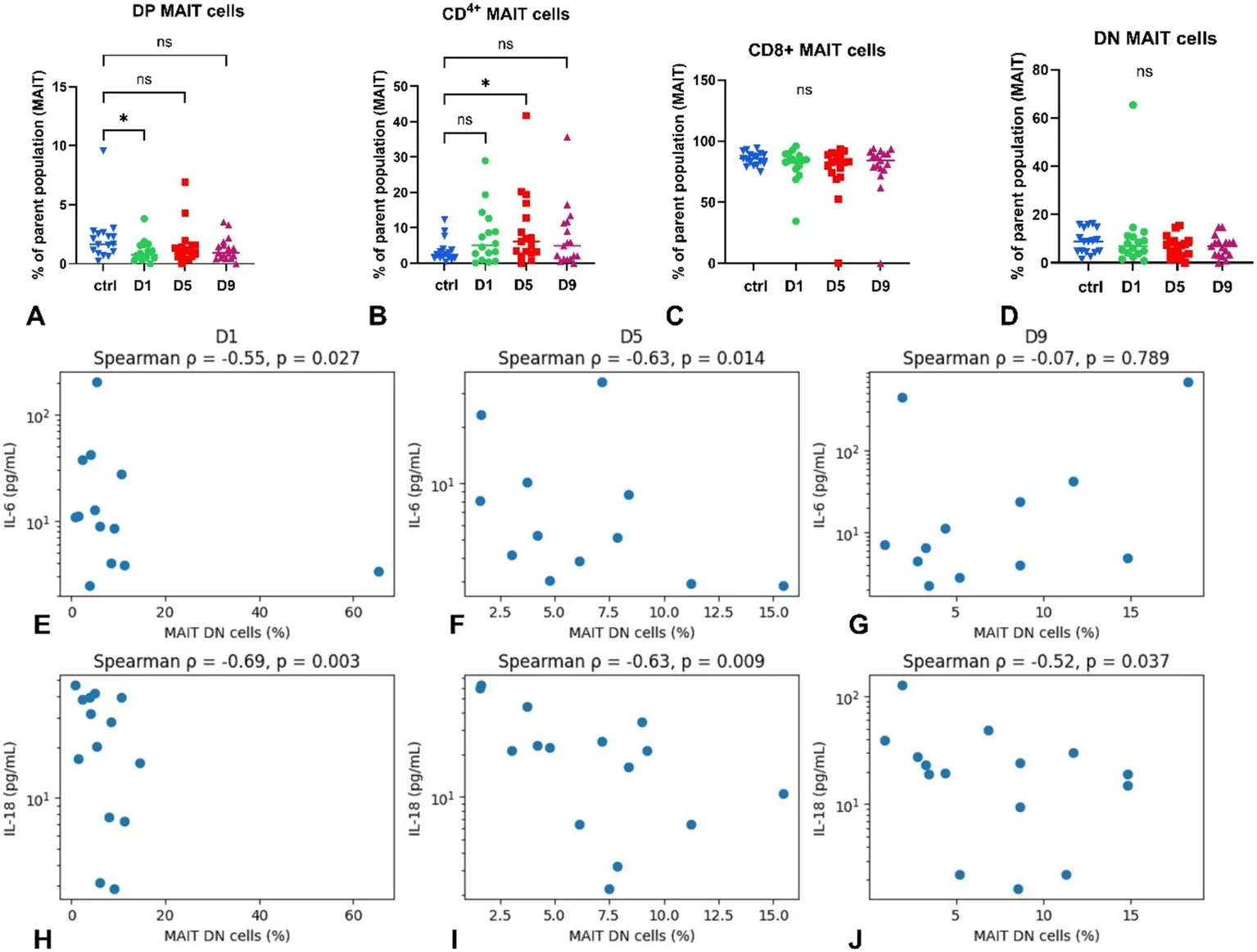
Distribution of MAIT cell subsets and their association with circulating cytokines following aneurysmal subarachnoid hemorrhage (aSAH). Flow cytometry was performed in a subset of patients with aSAH (*n* = 16) and compared to healthy controls (ctrl, *n* = 12). Peripheral blood samples were collected at day 1 (D1), day 5 (D5), and day 9 (D9) after ictus. MAIT cell subsets are expressed as percentages of the parent MAIT population. Data are presented as individual value plots, with each symbol representing one participant. **(A)** DP MAIT cells (double-positive; CD4^+^CD8^+^), **(B)** CD4^+^ MAIT cells, **(C)** CD8^+^ MAIT cells, **(D)** DN MAIT cells (double-negative; CD4^−^CD8^−^). Correlation analyses between DN MAIT cell frequencies and serum cytokine levels are shown for IL-6 at D1, D5, and D9 **(E–G)** and for IL-18 at D1, D5, and D9 **(H–J)**. Correlations were assessed using Spearman’s rank correlation coefficient (*ρ*), with corresponding *p*-values indicated in each panel. Statistical comparisons between groups (ctrl vs. D1, D5, D9) were performed using the Kruskal–Wallis test followed by Dunn’s multiple comparisons test. Significance levels are indicated as follows: NS (not significant), *p < 0.05, **p < 0.01.

Flow cytometric analysis of MAIT cell subsets and their association with circulating cytokines is shown in Figure 5. The proportion of DP (CD4^+^CD8^+^) MAIT cells was reduced at D1 compared to controls (*p* < 0.05), with no significant differences observed at D5 or D9 (Figure 5A). CD4^+^ MAIT cell frequencies were increased at D5 (*p* < 0.05), while no differences were detected at D1 or D9 (Figure 5B). In contrast, CD8^+^ MAIT cell proportions remained unchanged across all time points (Figure 5C), and no significant differences were observed for DN (CD4^−^CD8^−^) MAIT cells at any time point (Figure 5D). Correlation analysis revealed a negative association between DN MAIT cell frequencies and serum IL-6 levels at D1 (*ρ* = −0.55, *p* = 0.027) and D5 (ρ = −0.63, *p* = 0.014), while no correlation was observed at D9 (ρ = −0.07, *p* = 0.789; Figures 5E–G). For IL-18, DN MAIT cell frequencies also showed negative correlations at D1 (ρ = −0.69, *p* = 0.003), D5 (ρ = −0.63, *p* = 0.009), and D9 (ρ = −0.52, *p* = 0.037; Figures 5H–J). After Benjamini–Hochberg false discovery rate correction for the six correlation analyses, all significant correlations remained statistically significant (all *q* < 0.05), whereas the D9 IL-6 correlation remained non-significant.

Because delayed cerebral ischemia represents the major secondary complication after aSAH, additional exploratory analyses were performed according to DCI status. No significant differences in serum IL-6, IL-15, or IL-22 concentrations were observed between patients with and without DCI after Benjamini–Hochberg false discovery rate correction. Although IL-6 concentrations were nominally higher at Day 9 in patients who developed DCI (unadjusted *p* = 0.042), this association did not remain significant after correction for multiple comparisons.

Similarly, flow cytometric analyses revealed no significant differences in total MAIT-cell frequencies, DN MAIT cells, or other investigated lymphocyte populations according to DCI status after FDR correction. Total MAIT-cell frequencies were numerically lower in the DCI group at all three time points, but these differences did not reach statistical significance.

## 4 Discussion

In this study, we demonstrated a pronounced and sustained systemic inflammatory response following aneurysmal subarachnoid hemorrhage, characterized by persistently elevated circulating IL-6, IL-18, IL-15, IL-12p40, IL-22, and CXCL-9 levels, alongside later elevations of IL-7 and IP-10. While several cytokines were associated with clinical outcome in univariate analyses, these associations were attenuated after adjustment for WFNS score and age and did not remain statistically significant in exploratory penalized regression analyses. Therefore, our findings support the presence of sustained systemic inflammatory activation after aSAH rather than the identification of independent prognostic cytokine biomarkers. In parallel, flow cytometric analyses revealed persistent alterations in innate-like lymphocyte populations, most notably a reduction in circulating MAIT cells. Furthermore, DN MAIT cell frequencies showed significant negative correlations with serum IL-6 and IL-18 levels, linking cellular immune alterations with systemic inflammatory activation. These findings demonstrate concurrent alterations in circulating cytokine levels and innate-like lymphocyte populations following aSAH; however, the biological relationship between these observations cannot be established from the present study. These findings are consistent with previous reports indicating that both innate and adaptive immune pathways are activated after aSAH and contribute to secondary brain injury and systemic immune dysregulation (4, 6–8). Among the investigated cytokines, IL-6 showed the most prominent and persistent elevation across all examined time points. IL-6 is one of the best-characterized inflammatory mediators in aSAH and has repeatedly been associated with disease severity, cerebral vasospasm, delayed cerebral ischemia (DCI), and poor neurological outcome (17–19). Previous clinical studies demonstrated that elevated IL-6 concentrations in serum and cerebrospinal fluid occur early after hemorrhage and may remain increased during the acute neurocritical care period (17). Our findings of persistently elevated but temporally stable IL-6 levels between Day 1 and Day 9 are in line with these observations and further support the concept of sustained systemic inflammatory activation after aSAH rather than a transient cytokine response.

Similarly, IL-18 concentrations were markedly elevated throughout the examined period and closely correlated with IL-15 levels. IL-18 is a central inflammasome-associated cytokine implicated in sterile neuroinflammation following hemorrhagic brain injury (5). IL-18 may be released downstream of inflammasome activation and may contribute to amplification of sterile neuroinflammatory responses after SAH (20). The strong association observed between IL-18 and IL-15 in the present study may reflect coordinated activation of innate immune signaling pathways, as IL-15 is known to regulate NK-cell and memory T-cell activation during inflammatory conditions (21). Although IL-15 has been less extensively investigated in aSAH compared with IL-6, elevated IL-15 levels have been described in acute brain injury states and may contribute to prolonged immune activation and lymphocyte modulation (21).

In contrast to the persistently elevated IL-6/IL-18/IL-15 cluster, IL-7 and IL-12p40 exhibited distinct temporal patterns. IL-7 differed from controls only at Day 9, suggesting a later systemic elevation during the subacute phase after aSAH. In contrast, IL-12p40 was already elevated at Day 1 and remained significantly increased throughout the observation period, while longitudinal analysis demonstrated a further increase toward Day 9. IL-7 is a key regulator of T-cell homeostasis and lymphocyte survival, and delayed elevation of IL-7 may reflect compensatory immune restoration following early post-aSAH immunosuppression (22). Likewise, IL-12p40, which forms part of both IL-12 and IL-23 signaling pathways, has been associated with T-cell differentiation and chronic inflammatory activation (23). The observed correlations between IL-12p40, IL-7, and IL-22 suggest partially overlapping immune regulatory pathways during the subacute phase after hemorrhage, although the underlying biological relationships remain speculative.

Chemokine analysis demonstrated distinct temporal profiles for IP-10 and CXCL-9. IP-10 (CXCL-10) and CXCL-9 are interferon-inducible chemokines involved in the recruitment of activated T lymphocytes and NK cells through CXCR3-mediated signaling (24). Previous studies have demonstrated increased CXCL-10 expression after experimental and clinical SAH, where elevated levels were associated with leukocyte recruitment and inflammatory activation (24). Similarly, CXCL-9 has been implicated in CXCR3-mediated immune-cell trafficking (25). In the present study, IP-10 did not differ from controls at Day 1 but became significantly elevated at Days 5 and 9, whereas CXCL-9 was already elevated at Day 1, with more pronounced differences observed at Days 5 and 9. These findings suggest distinct temporal patterns of interferon-inducible chemokine responses following aSAH. The correlated clustering of IP-10 and CXCL-9 indicates that these chemokines may represent a partially distinct inflammatory module; however, the biological significance of this clustering and its relationship to immune-cell trafficking cannot be established from the present observational data.

Interestingly, IL-22 demonstrated only modest but consistently elevated levels throughout the examined period. IL-22 is increasingly recognized as a pleiotropic cytokine with context-dependent pro-inflammatory and tissue-protective effects (26). Although its role in aSAH remains poorly characterized, IL-22 has been implicated in regulation of endothelial integrity and tissue repair in inflammatory CNS disorders (26). The observed correlation between IL-22 and IL-12p40 may indicate shared regulation through Th17- and IL-23-related immune pathways, although the functional significance of this finding in aSAH requires further investigation. Overall, the observed cytokine patterns and hierarchical clustering analyses indicate the presence of multiple partially overlapping inflammatory profiles following aSAH, including persistent innate inflammatory activation, temporally distinct chemokine responses, and later changes in selected cytokines. However, these findings should be interpreted as associative and hypothesis-generating, and further mechanistic studies are required to determine the underlying biological pathways.

In our study, unfavorable clinical outcome after aSAH was associated with persistently elevated IL-6 and IL-15 levels, whereas IL-22 showed higher concentrations in patients with favorable recovery, although these associations were attenuated after adjustment for WFNS score and age and did not remain statistically significant in the exploratory Firth penalized regression models. Our findings are consistent with previous studies demonstrating that elevated systemic IL-6 levels after aneurysmal subarachnoid hemorrhage are associated with post-SAH complications, delayed cerebral ischemia, and unfavorable functional outcome, supporting the concept that persistent IL-6 elevation reflects the severity of post-hemorrhagic inflammatory activation (17). Our observation that persistently elevated IL-15 levels were associated with unfavorable outcome after aSAH is supported by accumulating evidence implicating IL-15 in cerebrovascular inflammation and hemorrhagic brain injury. Yang et al. (27) demonstrated significantly increased serum IL-15 levels in patients with intracranial aneurysms, with higher concentrations associated with aneurysm progression and inflammatory activation. More recently, Braun et al. (28) identified early alterations of multiple inflammation-related proteins in both cerebrospinal fluid and plasma following aneurysmal subarachnoid hemorrhage, supporting the concept of sustained systemic and central immune activation during the acute phase after hemorrhage. In experimental intracerebral hemorrhage, Shi et al. (29) further showed that IL-15 promoted astrocyte–microglia crosstalk, amplified neuroinflammatory signaling, and exacerbated neuronal injury, suggesting that increased IL-15 activity may contribute to secondary brain injury mechanisms after hemorrhagic cerebrovascular events.

Interestingly, IL-22 levels showed an opposite pattern compared with IL-6 and IL-15, with consistently higher concentrations observed in patients with favorable clinical outcome. Although the role of IL-22 in aneurysmal subarachnoid hemorrhage remains poorly characterized, previous studies suggest that IL-22 may exert context-dependent neuroprotective and tissue-protective effects following acute CNS injury. Dong et al. (30) demonstrated in a cerebral ischemia–reperfusion model that IL-22 attenuated inflammation, oxidative stress, and neuronal apoptosis through activation of the JAK2/STAT3 signaling pathway, thereby improving neurological injury. In parallel, recent reviews of neuroimmune responses after traumatic brain injury highlighted the complex immunomodulatory role of IL-22 in regulating tissue repair, inflammatory balance, and neuroimmune homeostasis following CNS damage (31). Therefore, while these experimental findings provide a plausible biological context for our univariate observations, the present clinical data do not support an independent prognostic role for IL-22, and the proposed mechanisms should be regarded as hypothesis-generating rather than directly demonstrated in patients with aSAH.

Importantly, linear mixed-effects analyses did not identify significant time-by-outcome interactions for IL-6, IL-15, or IL-22. Therefore, although concentrations differed between favorable and unfavorable outcome groups at individual time points, the temporal trajectories of these cytokines over the first 9 days were broadly parallel. This suggests that the observed outcome-related differences primarily reflected persistent between-group differences rather than distinct outcome-specific temporal cytokine responses.

One of the most notable findings of the present study was the persistent reduction of circulating MAIT-cell frequencies following aneurysmal subarachnoid hemorrhage. Although MAIT cells have not previously been extensively investigated in aSAH, accumulating evidence suggests that unconventional T-cell populations undergo substantial activation and redistribution during acute CNS injury. Pu et al. (32) demonstrated marked activation and functional alteration of MAIT cells in patients with intracranial infection, while Lv et al. (33) highlighted the broader role of unconventional T cells in neuroimmune regulation, cytokine signaling, and tissue trafficking during brain injury and neurodegeneration. In parallel, Roa et al. (8) reported dynamic alterations of peripheral immune-cell populations after aSAH, supporting the concept of extensive post-hemorrhagic immune remodeling.

In this context, the persistently reduced MAIT-cell frequencies observed in our cohort may reflect changes in peripheral MAIT-cell dynamics following aSAH, although the underlying biological mechanisms cannot be determined from the present data.

Because DCI represents the principal secondary neurological complication after aSAH, we additionally explored whether the observed immune alterations were associated with DCI development. Neither serum cytokine concentrations nor MAIT-cell frequencies differed significantly according to DCI status after correction for multiple testing. Although total MAIT-cell frequencies were consistently lower in patients who developed DCI, the flow cytometry subgroup included only five DCI cases and was therefore substantially underpowered to detect moderate between-group differences. Consequently, these exploratory analyses do not support a robust association between the investigated immune markers and DCI in the present cohort, but larger studies will be required to clarify this question.

Interestingly, although DN MAIT-cell proportions were not significantly altered compared with controls, they demonstrated strong negative correlations with circulating IL-18 levels across all examined time points, as well as with IL-6 during the early phase after hemorrhage. Since IL-18 is a potent inflammasome-associated cytokine capable of activating MAIT cells independently of T-cell receptor signaling (34), these findings suggest a potential association between inflammasome-driven systemic inflammation and MAIT-cell homeostasis after aSAH. Although the overall frequency of DN MAIT cells did not differ significantly between patients and controls, inverse correlations between DN MAIT-cell frequencies and circulating IL-18 (across all three time points) as well as early IL-6 remained significant after false discovery rate correction. These findings should not be interpreted as evidence of a causal relationship or selective depletion of the DN MAIT-cell subset. Rather, they suggest that inter-individual variation in DN MAIT-cell frequencies may be associated with the magnitude of systemic inflammatory activation and inflammasome-related immune responses following aSAH. Further studies incorporating functional characterization of MAIT cells will be required to clarify the biological significance of these associations.

### 4.1 Study limitations

Several limitations of the present study should be acknowledged. First, this was a single-center study with a relatively limited sample size, particularly in the flow cytometry subgroup, which included only 16 patients and 12 healthy controls. In addition, the flow cytometry subgroup represented a convenience sample determined by the availability of laboratory personnel and immediate PBMC processing facilities at the time of the initial blood collection. Although baseline clinical characteristics were comparable between the flow cytometry subgroup and the overall cohort, the possibility of selection bias cannot be completely excluded. Consequently, the cellular immune analyses should be considered exploratory and hypothesis-generating. In addition, flow cytometric analyses were performed on isolated PBMCs rather than whole blood. Consequently, only relative frequencies of lymphocyte subsets could be determined, whereas absolute circulating cell counts (cells/μL) were not available. Calculation of absolute counts would have required paired complete blood count measurements obtained from the same blood sample at each time point, which were not consistently available and were not collected in healthy controls. Therefore, we cannot completely exclude that changes in relative lymphocyte subset frequencies were influenced by alterations in overall leukocyte composition or hemodilution. However, because MAIT cells were quantified as a proportion of the CD3^+^ T-cell compartment and the proportion of total CD3^+^ T cells did not differ significantly between patients and controls (Figure 4F), the observed findings are not readily explained by changes in the overall T-cell compartment alone. Nevertheless, the absence of absolute cell counts precludes definitive exclusion of this possibility. The modest cohort size also limited the statistical power of multivariable analyses and restricted the number of confounders that could be included in regression models without risk of overfitting. Second, although longitudinal cytokine measurements were analyzed using linear mixed-effects models to account for repeated measurements, comparisons with healthy controls were performed at individual time points. Therefore, residual limitations related to repeated-measures analysis and multiple testing cannot be entirely excluded despite the application of a global Benjamini–Hochberg false discovery rate correction for patient-versus-control cytokine comparisons and mixed-effects modeling for longitudinal analyses. Third, outcome-associated cytokine differences observed in univariate analyses were attenuated after adjustment for WFNS score and age, indicating that these inflammatory markers are closely linked to initial disease severity. Additional clinically relevant confounders, including treatment-related variables, infectious complications, sedation, mechanical ventilation, or delayed cerebral ischemia, were not incorporated into multivariable models because of sample size limitations. Another limitation is the restricted temporal sampling window. Cytokine measurements and immune phenotyping were performed only during the first 9 days following ictus, preventing assessment of later immunological alterations during the subacute and chronic phases of recovery. Another limitation is the selection of the longitudinal sampling time points. Although an additional Day 3 sample might have provided greater temporal resolution during the transition from early brain injury to delayed secondary injury, the sampling schedule was determined by the practical constraints of a prospective longitudinal biobanking protocol. An initial five-time-point protocol (Days 1, 3, 5, 7, and 9) was found to be excessively demanding for critically ill patients and required substantial clinical and laboratory resources. Consequently, a three-time-point design (Days 1, 5, and 9) was adopted as a pragmatic compromise to ensure protocol adherence while supporting multiple concurrent translational studies using the same biospecimen collection. Similarly, only peripheral blood samples were analyzed; therefore, the relationship between systemic immune responses and local neuroinflammatory processes within the cerebrospinal fluid or central nervous system could not be evaluated. In addition, although significant alterations in MAIT cell frequencies were identified, the present study did not include functional characterization of these cells, such as cytokine production, activation status, exhaustion markers, or transcriptomic profiling. Therefore, the mechanistic role of MAIT cells in post-aSAH immune dysregulation remains to be clarified. In addition, detailed information regarding body mass index and regular medication use in healthy controls was not systematically collected, which may have introduced residual heterogeneity within the control cohort. Functional outcome was assessed at 3 months after aSAH. Although this is a widely accepted endpoint in cerebrovascular research, neurological recovery may continue beyond this period; therefore, future longitudinal studies incorporating 6-month or longer follow-up assessments may provide a more comprehensive evaluation of long-term functional outcome. Finally, due to the observational design of the study, causal relationships between cytokine alterations, immune cell dynamics, and clinical outcome cannot be established. Future multicenter studies with larger cohorts, extended longitudinal follow-up, and functional immune analyses will be required to validate and further define the immunological mechanisms identified in the present work.

## Data Availability

The original contributions presented in the study are included in the article/Supplementary material, further inquiries can be directed to the corresponding author.

## Supplementary material

SUPPLEMENTARY FIGURE S1 STROBE flow diagram of patient enrollment and longitudinal sample availability. September 2023 and July 2025, 90 consecutive patients presenting with subarachnoid hemorrhage were screened for eligibility. Forty-two patients were excluded because of late admission (*n* = 14), inability to obtain a Day 1 blood sample within the predefined sampling window (*n* = 6), non-aneurysmal (perimesencephalic) subarachnoid hemorrhage (*n* = 5), traumatic subarachnoid hemorrhage (*n* = 6), refusal to participate (*n* = 3), active infection (*n* = 3), autoimmune disease (*n* = 4), or chronic immunosuppressive therapy (*n* = 1). The remaining 48 patients fulfilled all eligibility criteria and were enrolled in the prospective study. No patients were lost during longitudinal follow-up, and complete serum samples were available from all participants at Days 1, 5, and 9. Flow cytometric analyses were performed in a predefined subgroup of 16 patients requiring immediate peripheral blood mononuclear cell processing. The healthy control cohort consisted of 20 individuals for cytokine analyses, including 12 participants who underwent flow cytometric analysis.
