# Supplementary material for: Longitudinal immune profiling demonstrates persistent MAIT cell reduction and temporal cytokine alterations after aneurysmal subarachnoid hemorrhage
Source: Front Neurol. 2026 Aug 20;17:1900084. doi: 10.3389/fneur.2026.1900084 (PMC13537967; doi:10.3389/fneur.2026.1900084)
Supplement: Supplementary file 1 [file Table_1.DOCX]

**Table S1. Benjamini–Hochberg False Discovery Rate (FDR)-Adjusted q-values for Patient-versus-Control Cytokine Comparisons**

Patient-versus-control comparisons were performed using two-sided Mann–Whitney U tests at each time point. Raw p-values from all 30 comparisons (10 cytokines measured at three time points) were adjusted simultaneously using the Benjamini–Hochberg false discovery rate procedure.

| Cytokine | Day 1 vs. Control | Day 5 vs. Control | Day 9 vs. Control |
| --- | --- | --- | --- |
| IL-6 | 0.0000018 | 0.0000018 | 0.0000034 |
| IL-7 | 0.3189 | 0.1931 | 0.00134 |
| IL-12p40 | 0.01457 | 0.00134 | 0.000237 |
| IL-12p70 | 0.5097 | 0.4203 | 0.4265 |
| IL-15 | 0.0000180 | 0.0000029 | 0.0000402 |
| IL-17A | 0.1688 | 0.7611 | 0.8456 |
| IL-18 | 0.0000029 | 0.0000029 | 0.0000045 |
| IL-22 | 0.00567 | 0.01322 | 0.01281 |
| IP-10 | 0.4152 | 0.01169 | 0.03022 |
| CXCL-9 | 0.03710 | 0.0000349 | 0.000197 |

**Table S2.** Benjamini–Hochberg false discovery rate (FDR)-adjusted q-values for patient-versus-control cytokine comparisons. Cytokine concentrations in patients with aneurysmal subarachnoid hemorrhage (aSAH) were compared with healthy controls at each time point (Day 1, Day 5, and Day 9) using two-sided Mann–Whitney U tests. To control for multiple testing, raw p-values from all 30 patient-versus-control comparisons (10 cytokines measured at three time points) were adjusted simultaneously using the Benjamini–Hochberg FDR procedure.
